# Surveillance for Avian Influenza A(H7N9), Beijing, China, 2013

**DOI:** 10.3201/eid1912.130983

**Published:** 2013-12

**Authors:** Peng Yang, Xinghuo Pang, Ying Deng, Chunna Ma, Daitao Zhang, Ying Sun, Weixian Shi, Guilan Lu, Jiachen Zhao, Yimeng Liu, Xiaomin Peng, Yi Tian, Haikun Qian, Lijuan Chen, Quanyi Wang

**Affiliations:** Beijing Center for Disease Prevention and Control, Beijing, China; Beijing Research Center for Preventive Medicine, Beijing;; Capital Medical University School of Public Health, Beijing

**Keywords:** influenza, avian influenza, avian influenza virus, viruses, influenza A(H7N9) virus, surveillance, Beijing, China

## Abstract

During surveillance for pneumonia of unknown etiology and sentinel hospital–based surveillance in Beijing, China, we detected avian influenza A(H7N9) virus infection in 4 persons who had pneumonia, influenza-like illness, or asymptomatic infections. Samples from poultry workers, associated poultry environments, and wild birds suggest that this virus might not be present in Beijing.

On March 31, 2013, three human infections with a novel avian influenza A(H7N9) virus were identified in Shanghai and Anhui Provinces in southeastern China (*1**,**2*). As of June 30, 2013, a total of 132 patients infected with this virus were reported in mainland China; 43 of these patients died (*3*). After detection of this novel virus, 6 targeted surveillance and sampling programs were implemented in Beijing, China, to identify possible cases.

## The Study

The study was approved by the Institutional Review Board of the Beijing Center for Disease Control and Prevention (CDC). During April 1–June 30, samples were collected during surveillance and sampling programs in Beijing. Patients with pneumonia of unknown etiology in all hospitals were reported to the correspondent district CDC. Respiratory specimens were collected and sent to the district CDC for testing for avian influenza A(H7N9) virus by real-time PCR. Suspected positive specimens were verified by the Beijing CDC.

Surveillance was conducted in 23 sentinel hospitals and 17 collaborating laboratories during April 22–June 30. Surveillance participants were defined as patients with influenza-like illness (ILI) ≤3 days after illness onset (*4**,**5*). Pharyngeal swab specimens were collected from surveillance participants and sent to collaborating laboratories. Specimens were first screened by real-time PCR to identify influenza A and B viruses. Samples positive for influenza A virus were tested for influenza A(H3N2) virus , influenza A(H1N1)pdm09 virus, and influenza A(H7N9) virus by real-time PCR. Specimens positive for seasonal influenza viruses were subjected to virus isolation.

Persons in close contact with patients infected with influenza A(H7N9) virus or infected animals were medically observed for 7 days after the most recent exposure during April 1–June 30. Pharyngeal swab specimens were collected from close contacts on each day during medical observation and tested for influenza A(H7N9) virus by real-time PCR.

Poultry workers from 5 districts in Beijing were sampled during April 19–28. Pharyngeal swab specimens were collected from poultry workers and sent to the correspondent district CDC for detection of influenza A(H7N9) virus by real-time PCR.

At the same time that poultry workers were sampled, environmental samples were collected from associated poultry environments. These environmental samples were sent to district CDC laboratories for detection of influenza A(H7N9) virus.

Samples were collected from wild birds in 317 parks and 10 wetland natural reserve regions in Beijing (Figure) during May 3–10. Ten fecal samples were collected from each park, and 20 fecal samples were collected from each region. Samples were sent to the correspondent district CDC laboratories for detection of influenza A(H7N9) virus.

**Figure:**
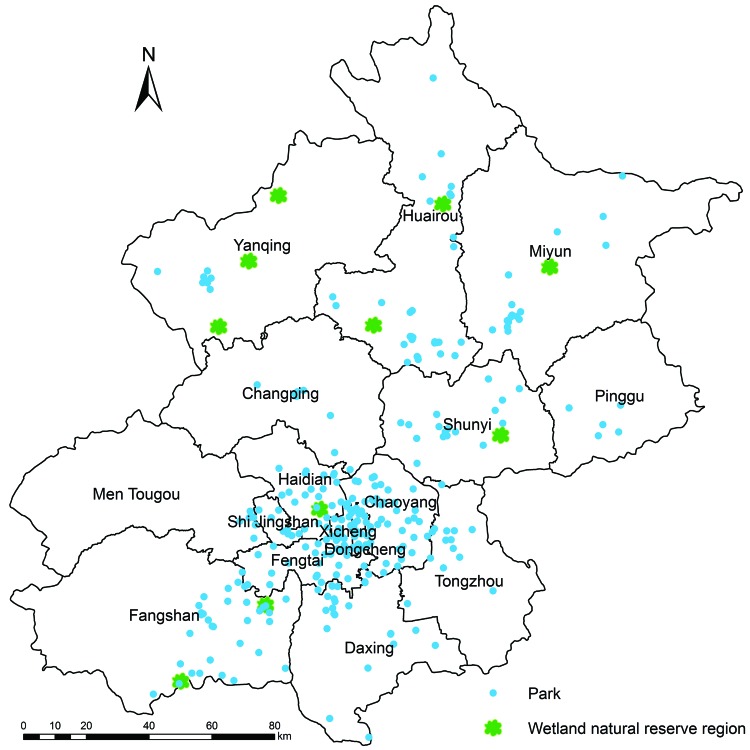
Distribution of 317 parks (blue dots) and 10 wetland natural reserve regions (green leaves) in which surveillance for avian influenza A(H7N9) virus was conducted, Beijing, China, 2013.

Thirty-nine patients with pneumonia of unknown etiology were reported, and 1 of these patients was infected with influenza A(H7N9) virus. This case was in a 7-year-old girl who had pneumonia, which was confirmed on April 12. The girl’s mother and a 4-year-old acquaintance (boy) were positive for influenza A(H7N9) virus but were asymptomatic. This cluster involved 2 families of chicken dealers who lived in Beijing. On April 4, both families had purchased live chickens from the same batch, which had been transported from Tianjin, China. Samples from environments of places of residence of both families were positive for influenza A(H7N9) virus.

In sentinel hospital and laboratory-based surveillance, 3,526 pharyngeal swab specimens were subjected to PCR. One specimen was positive for influenza A(H7N9) virus and 73 specimens were positive for seasonal influenza viruses. The patient infected with influenza A(H7N9) virus was a 6-year-old boy who resided in Beijing. On May 21, fever, sore throat, and headache developed in this patient. He visited a sentinel hospital during May 21–24 and recovered on May 23. He did not have pneumonia or any history of exposure to birds or other animals. He had visited the hometown of his family in Shandong Province during May 14–15.

A total of 1,422 poultry workers were recruited into this surveillance study, and 14 (0.98%) had ILI symptoms ≤2 weeks before recruitment. All workers were sampled but none were positive for influenza A(H7N9) virus. From the environments of poultry workers, 679 samples were collected; all were negative for influenza A(H7N9) virus. However, 3 samples from 2 other locations were positive for influenza A(H9N2) virus. In addition, 3,401 fecal samples of wild birds were collected; all were negative for influenza A virus.

## Conclusions

Clinical manifestations of human infections with influenza A(H7N9) virus found in Beijing included pneumonia, ILI, and asymptomatic infection. Although patients infected with this virus in China have so far had lower respiratory tract infections (*6**,**7*), our findings suggest that infection with influenza A(H7N9) virus could cause a wide spectrum of clinical illness.

The first patient infected with influenza A(H7N9) virus in Beijing was found by surveillance for pneumonia of unknown etiology, which was initially designed for finding patients with severe acute respiratory syndrome or influenza A(H5N1) virus infection. Although hospital-based surveillance is less efficient in finding mild and asymptomatic infections, it may be the most feasible approach for identifying severe cases of infection with influenza A(H7N9) virus.

The second patient infected with influenza A(H7N9) virus, the 6-year-old boy, was found through sentinel hospital and laboratory-based surveillance. Before emergence of influenza A(H7N9) virus, surveillance was conducted by using cell culture–based virus isolation techniques. To increase assay sensitivity and rapidity, real-time PCR preceding virus isolation was adopted to replace the strategy of only using virus isolation. In addition, because only specimens positive for seasonal influenza viruses were subjected to virus isolation, this procedure could help avoid the risk for propagating influenza A(H7N9) virus from unknown specimens, as is caused by conducting virus isolation directly in biosafety level 2 laboratories. Our findings and those of another report (*8*) demonstrated that patients infected with influenza A(H7N9) virus only had ILI. Therefore, the strategy of PCR preceding virus isolation should be the preferred option during sentinel hospital and laboratory-based surveillance.

Live poultry is the major source of avian influenza A(H7N9) (*6**, **9**–**11*). In addition, migratory birds may participate in multiple reassortment events for emergence of H7N9 subtype virus (*1**,**12*). Therefore, the role of wild birds in transmission of avian influenza A(H7N9) virus to poultry or humans should not be ignored. In our samples from poultry workers, associated poultry environments, and wild birds, influenza A(H7N9) virus was not found, which suggests that this virus might not be present in Beijing.

In conclusion, human infections with H7N9 virus can cause a wide spectrum of clinical illnesses. Surveillance of patients with pneumonia of unknown etiology is preferred for early detection of severe cases. PCR is recommended for screening in sentinel hospital and laboratory-based surveillance of influenza A(H7N9).
